# Selective mortality in middle-aged American women with Diffuse Idiopathic Skeletal Hyperostosis (DISH)

**DOI:** 10.1371/journal.pone.0202283

**Published:** 2018-08-28

**Authors:** George R. Milner, Jesper L. Boldsen, Stephen D. Ousley, Sara M. Getz, Svenja Weise, Peter Tarp, Dawnie W. Steadman

**Affiliations:** 1 Department of Anthropology, Pennsylvania State University, University Park, Pennsylvania, United States of America; 2 Unit of Anthropology, ADBOU, Department of Forensic Medicine, University of Southern Denmark, Odense, Denmark; 3 Department of Mathematics and Information Science, Mercyhurst University, Erie, Pennsylvania, United States of America; 4 Department of Anthropology, Idaho State University, Pocatello, Idaho, United States of America; 5 Forensic Anthropology Center, Department of Anthropology, University of Tennessee, Knoxville, Tennessee, United States of America; Medical College of Wisconsin, UNITED STATES

## Abstract

**Objective:**

A mortality sample of white American male and female skeletons was examined to illustrate a simple means of identifying skeletal conditions associated with an increased risk of dying relatively early in adulthood and to determine if males and females with Diffuse Idiopathic Skeletal Hyperostosis (DISH) displayed the same general age-specific pattern of mortality.

**Methods:**

Age-specific probability distributions for DISH were generated from 416 white Americans who died from the 1980s to the present, and whose remains were donated to the University of Tennessee Forensic Anthropology Center. The age-specific frequency of DISH is analyzed using an empirical smoothing algorithm. Doing so allows for the identification of deviations (i.e., local maxima) from monotonically increasing age-specific probabilities.

**Results:**

In females (N = 199), there is a peak in the frequency of individuals with DISH around 60 years of age where 37.0% of the individuals have DISH. It is matched only by the frequency (38.7%) in the oldest females, those over 85 years old. In contrast, DISH frequencies for males (N = 217) increase monotonically with advancing age, reaching 62.5% in the ≥86 years age group. There was an association between DISH and high body weight in women, particularly those who died before they reached the age of 75.

**Conclusions:**

Early-onset DISH in white American women is associated with an increased risk of dying indicated by a local maximum in the probability curve. Should this finding be replicated in additional mortality samples and the reason DISH is associated with early death is established, beyond being heavy, this radiologically visible ossification of the spine could be a potential component of health-monitoring programs for middle-aged women.

## Introduction

Diffuse Idiopathic Skeletal Hyperostosis (DISH), also called Forestier’s disease or ankylosing hyperostosis, among other names, is a systemic condition of unknown etiology featuring an exuberant ossification of ligaments and entheses, which is often widespread throughout much of the adult skeleton [1–7]. In modern populations, DISH occurs more commonly in males and in individuals over about 50 years old, it is found in people of different ancestries, and it is associated with some physical impairment [8–20]. A bony involvement consistent with DISH has also been described in archaeological skeletons, some of which date back as much as several thousand years [21–52]. The most apparent aspect of this condition, in both patients and skeletal remains, is a distinctive ossification of ligaments that unite adjoining vertebrae, leading to a spine lacking flexibility in part or all of its extent. In dry bones, spinal segments affected by DISH appear as if dense, smooth-surfaced bone had flowed, much like candlewax, over the anterior and lateral surfaces of vertebral bodies.

A mortality effect for DISH, suggested decades ago [11], has not yet been convincingly demonstrated [53], despite the ossification being reported as associated with several morbid conditions. Spinal fractures are an exception insofar as they are related to a rigid spine and an increased risk of fracture [54–58]. The value of skeletal (mortality) samples for identifying bony indicators of selective mortality is demonstrated by contrasting dissimilar age distributions for men and women with DISH in a skeletal collection from the United States. The objective is to identify in a mortality sample deviations from monotonically increasing DISH frequencies with advancing age. In clinical studies, such increases have been assumed and have been modeled accordingly for living subjects from the Netherlands [20]. The limited documentation available on the skeletal sample is examined to determine if there is any association between evidence for selective mortality and life histories, notably weight.

## Materials and methods

Modern American skeletons of known age and sex were examined for DISH. Age-specific probabilities of having DISH, generated from this mortality sample, were used to determine if the distinctive ossification of the spine was associated with an increased risk of dying relatively early in adulthood.

### Skeletons and DISH

Males (N = 217) and females (N = 199) in the Bass Donated Collection at the University of Tennessee Forensic Anthropology Center (FAC) [59] were examined for DISH while data were collected for a separate project designed to refine skeletal age-estimation procedures for adults from forensic contexts. The individuals, all of whom were at least 19 years old, were classified as white, and they had died from the 1980s to the present. For this group, the sample approximates a cross-sectional study of deaths. The skeletons were donated to the FAC for research purposes, typically forensic-related studies. They were not specially selected for their medical or occupational histories, or the cause of death.

This report on DISH is part of a larger study of skeletal changes associated with adult aging where the targeted sample was approximately 400 individuals from different continents. The American skeletal collection was stratified by age, using 10-year categories, to obtain a sample with individuals distributed across the adult lifespan. To do so, skeletons were randomly chosen from age categories that included numerous individuals. All individuals who died during the first and last several decades of adulthood were selected because there were fewer of them in the collection. The resulting age-at-death distribution, therefore, does not approximate what might be expected from the normal accumulation of deaths. That does not matter here because the concern is with the probability of having DISH at each age.

No skeleton in this study’s sample, all of which were chosen prior to collecting data, was excluded because of the individual’s age, sex, or, when available, reported weight, occupation, or cause of death. Skeletons were for the most part examined by their order of acquisition, which was how they were organized on shelves, and all information on storage boxes, except the skeleton designation, was hidden from view well in advance of data collection. Data on skeletal characteristics, including DISH, were collected blindly with respect to individual characteristics, notably age and sex, but also information on life and medical histories. Vertebrae broken postmortem or those misshapen by major antemortem trauma, such as healed compression fractures, were not scored for DISH.

Vertebral ossification scored as DISH was consistent with what has been described in studies of radiographs, CT scans, and dry bones [1, 2, 4–7, 12, 38, 46, 47, 60]. There is, however, a lack of consensus about the extent of bony involvement required for a diagnosis of DISH. Here only individuals with four or more adjacent vertebral bodies joined by bony bridging or interlocking masses of bone that immobilized joints were scored as positive for DISH. The multi-vertebrae criterion was consistent with many studies of patients and skeletons [5, 60–62].

The association between DISH and both poor health and weight were examined for males and females. Poor health was defined as having one or more of the following conditions listed in documentation, primarily cause of death, that accompanies the skeletons: cardiovascular disease, diabetes, and obesity [4, 7, 9, 12, 63–73]. These conditions were combined because when considered separately the samples were too small for analytical purposes. Weight was used alone, instead of being combined with stature to estimate BMI. There were too few individuals with both recorded weight and height for analytical purposes.

### Statistical methods

Age-specific probabilities of having DISH for each sex are estimated using two methods: linear logistic regression (through a generalized linear model, glm) and empirical smoothing (through a generalized additive model, gam). The R statistical software (version 3.2.3, dated 12/10/2015) was used for both estimation processes, with the R Library mgcv (version 1.8–9) for the gam [74–76]. A closer fit to data is potentially possible with a gam if the data, in this instance age-specific probabilities of having DISH, are distributed in an irregular enough fashion that they cannot be readily approximated by a simple parametric model. The Akaike information criterion (AIC) was used to identify curves that describe the data most parsimoniously [77].

The relationship between DISH and both body weight and the combined cardiovascular disease, diabetes, and obesity category were analyzed through Fisher’s Exact Tests using SPSS software (version 24). Heavy individuals were identified as those over 104.5 kg, which was approximately one standard deviation from the female and male means (138 females, mean 74.3 kg, standard deviation 29.9; 149 males, mean 82.1 kg, standard deviation 22.8). All individuals reported as obese in the available medical documentation were included in the heavy body weight category. Many heavy individuals as defined here, however, were not recorded as being obese.

## Results

**Fig 1:**
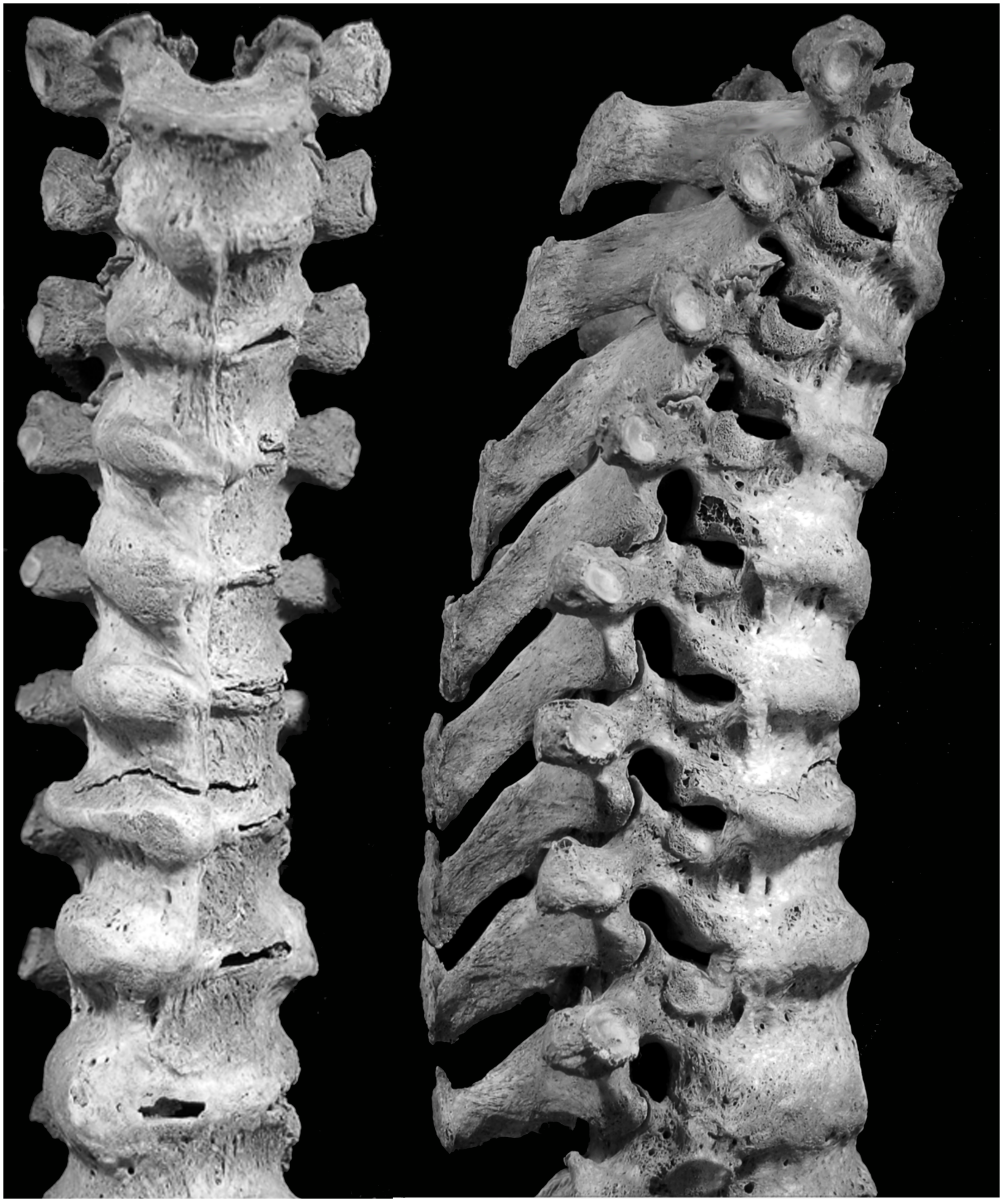
Diffuse Idiopathic Skeletal Hyperostosis (DISH). Vertebrae from a male, 81 years old, with the distinctive DISH ossification resembling candlewax.

Of 217 male and 199 female skeletons, 61 (28.1%) males and 44 (22.1%) females were scored positively for DISH (Table 1). These skeletons had a proliferation of smooth-surfaced bone on the anterolateral surfaces of normally shaped vertebral bodies (Fig 1). This ossification had a distinctive appearance, as if it had flowed like candlewax from one vertebra to the next; it was located on vertebral bodies, typically extending from the superior to inferior margins of vertebrae; a proliferation of bone was generally most pronounced where the anterior longitudinal ligament was located in life; and often it partly or completely bridged spaces between adjacent vertebrae, immobilizing them. Bony bridging protruded anteriorly and laterally, forming prominent bulges along the spine, and spaces where the intervertebral discs were once located were spared. On thoracic vertebrae, there was a tendency for more pronounced ossification on the right side.

**Table 1:** DISH in the American skeletal sample.

Age	Male			Female		
	N^a^	DISH^b^	DISH%^c^	N^a^	DISH^b^	DISH%^c^
≤45	66	2	3.0	29	0	0.0
46–55	29	3	10.3	32	2	6.3
56–65	25	7	28.0	27	10	37.0
66–75	31	14	45.2	37	8	21.6
76–85	42	20	47.6	43	12	27.9
≥86	24	15	62.5	31	12	38.7

^a^ Sample size

^b^ DISH cases

^c^ Percent with DISH

Of the DISH cases, the ossification occurred most often in the thoracic part of the spine where vertebrae were affected in 95.2% of 104 observable spinal segments. Cervical and lumbar vertebrae were involved in 17.5% of 103 and 41.3% of 104 observable spinal segments, respectively.

**Fig 2:**
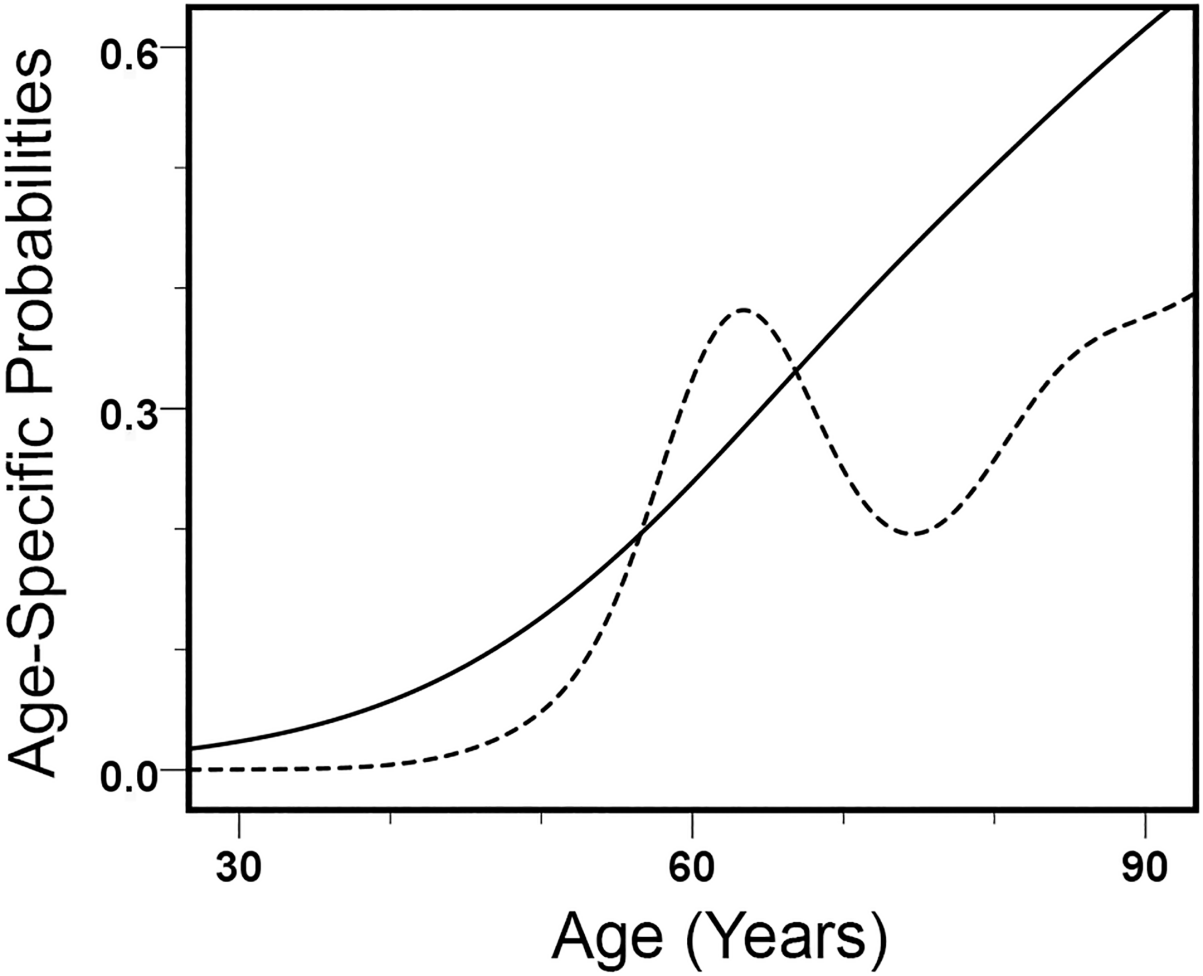
Probability curves for females and males with DISH. Female (dotted) and male (solid) probability curves for individuals with DISH, estimated from an American mortality sample through empirical smoothing. Male frequencies increase with advancing age. The female curve, with a peak around 60 years, indicates that women with early-onset DISH experience a relatively high risk of dying around that age.

In the American mortality sample, DISH probabilities increase monotonically in men, but not in women (Fig 2). For males, an empirically smoothed transition curve describing the trend in data is essentially indistinguishable from a linear logistic regression model (logistic AIC = 207.73, empirically smoothed AIC = 206.87). For women, the empirical smoothing algorithm, which results in a sinuous curve with a local maximum and a local minimum, provides a much better fit to the data than the simpler approach using linear logistic regression (logistic AIC = 196.83, empirically smoothed AIC = 189.95). There is an early peak for women around 60 years (a local maximum), followed by a trough in the 70s (a local minimum), and a final monotonous increase in old age.

The percentage of females with DISH in the 56–65 age group, 37.0%, is about as high as it is for those in the ≥86 group, 38.7% (Table 1). Male DISH frequencies in the age intervals used here exceed those for women in all corresponding categories, except the female peak at 56–65 years.

There was a strong association between early-onset DISH in women and body weight, but not the poor health category (any combination of recorded cardiovascular disease, diabetes, and obesity). The female probability distribution, with a local minimum around 75 years, was used to distinguish early and late mortality groups for both women and men (Fig 2). For the early mortality group, a lower limit was fixed at 46 years to include all women with DISH and to exclude those who were not at risk of death because of their young age (≤45 years). For the poor health category, individuals with DISH were not statistically different from those without DISH (*p*>0.05). Women with early-onset DISH, however, were heavier than those in the same age group who did not have the condition (*p* = 0.002) (Table 2). While old women with DISH were also heavier than those without it, the difference between them and lighter women was not as strong (*p* = 0.035). In men, weight and DISH approached statistical significance in the young age category (*p* = 0.064) but not the old one (*p* = 0.606).

**Table 2:** DISH and weight in the American skeletal sample.

Age	Sex	No DISH		DISH		FET^a^
		Light	Heavy	Light	Heavy	
46–75	Female	69	13	4	7	*p* = 0.002
46–75	Male	70	8	13	5	*p* = 0.064
≥76	Female	37	0	16	3	*p* = 0.035
≥76	Male	20	1	18	3	*p* = 0.606

^a^ Fisher’s Exact Test

## Discussion

### Identifying DISH

Criteria used to identify DISH are not consistent across studies, including clinical research [4, 61, 62], although the involvement of four adjacent vertebrae (three joints) is commonly used and is consistent with the original definition based on radiographic findings [5, 78]. The appearance of the ossification classified as DISH in the American skeletal sample is similar to what has been described in studies of patients and skeletons [1, 2, 4–7, 12, 38, 46, 47, 60]. On thoracic vertebrae, there was a tendency for more a pronounced involvement of the right side, consistent with clinical and anatomical studies where side asymmetry is attributed to the location of the descending aorta [4, 5, 7, 61, 78–80]. The thoracic region of the spine was more likely to be affected by DISH than cervical or lumbar vertebrae, similar to studies of living subjects where DISH commonly occurs in this part of the vertebral column, notably in the more caudally located thoracic vertebrae [5–7, 10, 13, 18, 58, 78].

When examining only bones without the benefit of the clinical aspects of diseases, the problems of correctly, or at least consistently, identifying the conditions of interest are increased. In this study, the pathological bony involvement classified as DISH might be confused with other conditions, including ankylosing spondylitis and advanced osteophytic development. However, the possibility of misclassification is diminished by the ossification’s location, extent, and appearance, specifically its resemblance to flowing candlewax, its smooth dense surface, and the number of contiguous vertebrae affected [79, 81, 82]. The comparatively low prevalence of ankylosing spondylitis relative to DISH [55, 81, 83, 84], further reduces the possibility that misdiagnoses, if they occurred, had a noticeable effect on our findings. Furthermore, there is no reason to believe the principal feature of interest, the spike in the female frequency distribution around 60 years, resulted from misclassification error concentrated in one sex and age group. That is because data were collected without knowledge of an individual’s age or sex.

The ossification criteria used here—four or more adjacent vertebrae affected—yielded lower frequencies than would be produced by other means of scoring DISH in skeletal remains [25, 46]. That is a desirable feature of the scoring procedure employed here. Conservative recognition criteria reduce the likelihood that other conditions might contribute to the irregularity in the female frequency distribution attributed to early-onset DISH.

### Frequency distributions

In a population homogeneous with respect to the aging process, it can be expected that the probability of having the condition increases monotonically with age if three conditions are met. First, having the condition is a function of age. Second, the condition, once developed, will not disappear. Third, the selective mortality effect is weak or absent. In situations where all three are in effect, frequencies in a mortality sample will simply increase with advancing age. The increase with age in a mortality sample will approximate what is found in a sample of living people. For patients, the probability of having developed DISH has been estimated for a sample from the Netherlands [20], and an increase with age is consistent with what was found here for males in the American mortality sample.

Irregular curves featuring peaks or plateaus can indicate a heterogeneous population. Imagine a situation where an observable bony characteristic develops relatively early in life and it is associated with an elevated risk of dying. Many other people who acquire the condition do so only later in life, and they suffer little in the way of related ill effects that lead to death. When all of these individuals are combined in a single age-specific frequency distribution, a peak in mortality corresponds to the years when many in the high-risk early-onset group died. The American mortality sample females are an example of such a frequency distribution. A high frequency of DISH in women who were about 60 years of age when they died is followed by a decline in DISH frequencies and, eventually, an increase in old age.

What happens to living individuals can be quite different from patterns detectible in skeletal samples. For each person when alive, the probability of having DISH increases with advancing age because the length of the period during which the ossification can develop also increases as the individual becomes older. What can be seen in a mortality sample, even though it includes the very same individuals, is different. Here selective mortality plays a part in the composition of the sample. For skeletal conditions associated directly or indirectly with a greater risk of dying, the age distribution of affected individuals in a mortality sample might not monotonically increase. That is because people with the condition of interest are preferentially selected out of their cohort, with the remainder, many of whom do not have the skeletal indicator of interest, living to an older age. The distribution of DISH by age for women in the American mortality sample indicates an early onset of the condition is likely to be associated with higher mortality. That would lead to a relatively early accumulation of deaths among women with DISH, in this instance around 60 years of age.

This work with American skeletons, where the first male with DISH was 39 years old and the first female was 49, shows that it is important to include young individuals in analyses of skeletal traits usually associated with old age. In that sense, it is similar to the findings of a study of age-specific prevalence of DISH in living subjects [20].

Early-onset DISH mortality in white American women is not the only occasion where deviations from monotonically increasing frequencies have been reported. For example, two recent surveys of Japanese patients found that the frequencies of females with DISH increased to the 70s, and then decreased [10, 17]. Male frequencies increased with advancing age in one study, and in the other there was a decline in the 90s, the terminal age interval. Such trend irregularities could result from inadequate sampling, but the possibility of selective mortality in connection with DISH deserves further attention.

### Advantages of skeletal collections

There are two major reasons the DISH frequency data presented here are not directly comparable to what is reported in the clinical literature. First, what can be observed in radiographs of living subjects is not the same as what is visible when looking directly at dry bones [85]. There is also interobserver variation in the identification of DISH when patients are evaluated with radiographs and CT scans [12, 86, 87]. This source of error, however, is much reduced in skeletal samples where direct examinations of bony structures are possible. Second, the samples are inherently different, with living subjects not being the same as skeletons. If there are no differences in the ability to detect an acquired pathological condition, frequencies derived from a mortality sample, such as a skeletal collection, will exceed those in the corresponding living sample, as long as the condition of interest is associated with a greater risk of dying [88, 89].

Because morphological changes in bone, even subtle ones, can be readily seen in dry, clean specimens, many aspects of skeletal aging can be easily detected in mortality samples. In the absence of selective mortality, for old people the probabilities associated with having such skeletal traits will be greater or equal to those for young ones. That is, the probability of having acquired the bony characteristic will be a non-declining function of age. A plateau in the mortality sample probability distribution for a trait immediately followed by a resumption of increasing probabilities indicates heterogeneity, but it does not necessarily involve mortality. The plateau in the probability distribution can be a consequence of heterogeneity in the skeletal aging process unrelated to the risk of dying. In contrast, an outright peak in the distribution of probabilities, arranged by age, in a mortality sample—younger individuals are more likely to display the trait than some, or all, older ones—can be an efficient means of identifying skeletal characteristics with a potential to serve as indicators of an increased risk of dying.

Another advantage of skeletal collections is they are likely to be unbiased samples with regard to pathological conditions that complicate DISH frequency estimates. Clinical samples such as hospitalized patients, in contrast, might include people with back pain, spinal fractures, or other debilitating conditions associated with DISH. There is no reason to suspect such sample-selection biases contributed to the relatively high risk of dying among women with early-onset DISH in the American mortality sample.

### Study limitations

Benefits from examining skeletons, rather than patients, are balanced by disadvantages that include the use of small mortality samples and limited life-history documentation. Skeletons, of course, are from people who failed to survive to an older age. The frequencies of bony features associated with a greater risk of dying are not the same as the prevalence of those same conditions in the living population from which the skeletons were derived [88, 89]. Frequencies of DISH cases at each age, therefore, are not directly comparable in patient and skeletal samples.

Although there are a number of known-age skeletal samples distributed around the world, including several in North America, obtaining large samples will always be difficult, especially when compared to the opportunities available for examining living subjects. In fact, this study’s sample is large for work with known-age skeletons, although its composition limits the findings to white Americans.

A sample consisting of only a few hundred skeletons coupled with sparse life history documentation prevents controlling for differences among individuals, including varied disease experience, other than by sex. Skeletal collections in general are of limited use when identifying what might lead to higher mortality among people with a pathological indicator of interest. This study’s sample is no exception to the rule.

Little more can be said than the elevated risk of dying associated with, as distinct from caused by, DISH that develops early in adulthood in American women must be related to something more serious than back stiffness and pain, no matter how discomforting. An ankylosed spine with compromised flexibility can be more susceptible to fracture, especially in the cervical region, accompanied by complications that include instability and spinal cord injury, and an elevated relative risk of dying [54, 55, 57, 58, 90–93]. The spike in DISH frequencies in women around 60 years of age in the American mortality sample, however, cannot be attributed to life-threatening spinal injuries. Fractures, especially those severe enough to be fatal, would not have occurred often enough to have such a noticeable effect on the female mortality distribution. Furthermore, the experience of men and women with regard to such injuries is unlikely to have been as dramatically different as indicated by the two mortality curves.

Connections have been drawn, however, between DISH and several conditions associated with a greater relative risk of dying, including obesity, diabetes, cardiovascular disease, and stroke, among others [4, 7, 9, 12, 63–73, 94]. Such potentially life-threatening conditions might lie behind the female mortality spike in the American sample. Among these deceased people, high body weight and DISH are more closely associated in females than in males, and in young than in old people. A statistically significant relationship between DISH and poor health, which includes documented cardiovascular disease, diabetes, or obesity, was not detected. This study, however, is not a good indication of a possible association between DISH and either cardiovascular disease or diabetes. Obesity, occasionally recorded with cause of death information, is captured by the more inclusive heavy body weight category used here. In the best of circumstances, reported cause of death is a poor indicator of chronic conditions that could be associated with DISH. Small samples, once the skeletons were divided by age, sex, and medical information, further diminish anything useful that could be said about DISH from available documentation.

## Conclusions

A mortality effect for DISH, suggested decades ago [11], has been identified for white American women with early-onset DISH. It is apparent as a peak in age-specific DISH frequencies in a skeletal (mortality) sample. The spinal ossification is a marker of increased risk, not a direct cause of death. Determining what contributes to that elevated risk of dying necessitates subsequent clinical studies, although early-onset DISH in American females is associated with being heavy. The mortality effect identified in this skeletal sample raises the possibility that DISH, detectable radiographically, can be a useful part of health-monitoring programs for middle-aged women.
